# [μ-2-(4-Hy­droxy­phen­yl)acetato]-κ^3^ *O*:*O*,*O*′;κ^3^ *O*,*O*′:*O*′-bis­{aqua­(4,4′-bi­pyridine-κ*N*)bis­[2-(4-hy­droxy­phen­yl)acetato-κ^2^ *O*,*O*′]holmium(III)} mono­hydrate

**DOI:** 10.1107/S1600536811001115

**Published:** 2011-01-15

**Authors:** Jia-Lu Liu, Jian-Feng Liu, Guo-Liang Zhao

**Affiliations:** a College of Chemistry and Life Sciences, Zhejiang Normal University, Jinhua 321004, People’s Republic of China and, Zhejiang Normal University Xingzhi College, Jinhua 321004, People’s Republic of China

## Abstract

In the title dinuclear complex, [Ho_2_(C_8_H_7_O_3_)_6_(C_10_H_8_N_2_)_2_(H_2_O)_2_]·H_2_O, each of the two independent Ho^III^ ions is coordinated by eight O atoms from four 4-hy­droxy­phenyl­acetate (HPAA) ligands and a water mol­ecule, and one N atom from a 4,4′-bipyridine (bipy) ligand in a distorted tricapped trigonal–prismatic geometry. The HPAA ligands are coordinated in bis-chelate, bridging and bridging tridentate modes. In the crystal, O—H⋯O and O—H⋯N hydrogen bonds link the mol­ecules into a three-dimensional network.

## Related literature

For background to the importance of coordination modes in magnetic structures, see: Fang & Zhang (2006 ▶); Munoz *et al.* (2005 ▶); Wang & Sevov (2008 ▶); Wang *et al.* (2010 ▶). For a related structure, see: Liu *et al.* (2010 ▶).
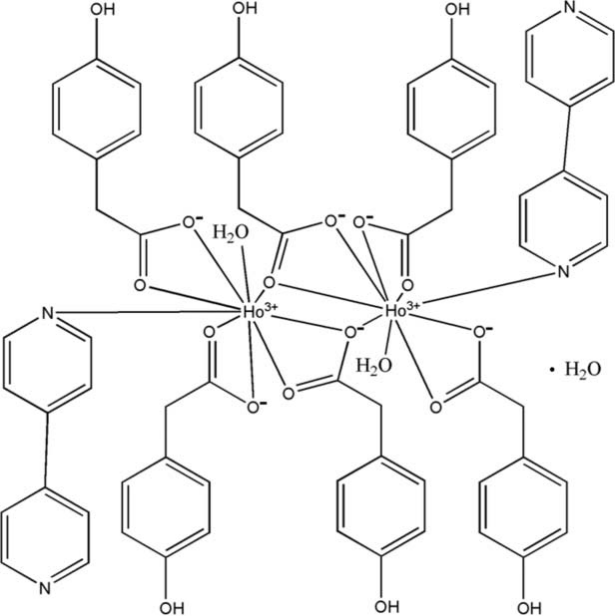


## Experimental

### 

#### Crystal data

[Ho_2_(C_8_H_7_O_3_)_6_(C_10_H_8_N_2_)_2_(H_2_O)_2_]·H_2_O
*M* *_r_* = 1603.09
Triclinic, 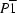

*a* = 11.7217 (7) Å
*b* = 16.1885 (9) Å
*c* = 18.413 (1) Å
α = 83.441 (3)°
β = 72.208 (3)°
γ = 71.337 (3)°
*V* = 3151.4 (3) Å^3^
*Z* = 2
Mo *K*α radiation
μ = 2.58 mm^−1^
*T* = 296 K
0.34 × 0.11 × 0.03 mm

#### Data collection

Bruker APEXII CCD diffractometer
Absorption correction: multi-scan (*SADABS*; Sheldrick, 1996 ▶) *T*_min_ = 0.469, *T*_max_ = 0.924
49489 measured reflections
14593 independent reflections
12826 reflections with *I* > 2σ(*I*)
*R*_int_ = 0.021

#### Refinement

*R*[*F* ^2^ > 2σ(*F* ^2^)] = 0.019
*wR*(*F* ^2^) = 0.051
*S* = 1.00
14593 reflections
874 parameters
9 restraints
H atoms treated by a mixture of independent and constrained refinement
Δρ_max_ = 0.58 e Å^−3^
Δρ_min_ = −0.66 e Å^−3^

### 

Data collection: *APEX2* (Bruker, 2006 ▶); cell refinement: *SAINT* (Bruker, 2006 ▶); data reduction: *SAINT*; program(s) used to solve structure: *SHELXS97* (Sheldrick, 2008 ▶); program(s) used to refine structure: *SHELXL97* (Sheldrick, 2008 ▶); molecular graphics: *SHELXTL* (Sheldrick, 2008 ▶); software used to prepare material for publication: *SHELXTL*.

## Supplementary Material

Crystal structure: contains datablocks I, global. DOI: 10.1107/S1600536811001115/lx2179sup1.cif

Structure factors: contains datablocks I. DOI: 10.1107/S1600536811001115/lx2179Isup2.hkl

Additional supplementary materials:  crystallographic information; 3D view; checkCIF report

## Figures and Tables

**Table 1:** Hydrogen-bond geometry (Å, °)

*D*—H⋯*A*	*D*—H	H⋯*A*	*D*⋯*A*	*D*—H⋯*A*
O6—H6*B*⋯O3*W*^i^	0.82	1.86	2.646 (3)	160
O9—H9*A*⋯O17^ii^	0.82	1.86	2.676 (2)	173
O12—H12*A*⋯O11^iii^	0.82	1.94	2.748 (2)	168
O15—H15*C*⋯O6^iv^	0.82	1.90	2.714 (2)	174
O18—H18*B*⋯O9^i^	0.82	1.95	2.769 (3)	174
O1*W*—H1*WA*⋯O13	0.84 (4)	1.95 (2)	2.736 (2)	156 (3)
O1*W*—H1*WB*⋯N4^v^	0.83 (2)	1.97 (2)	2.781 (3)	167 (4)
O2*W*—H2*WA*⋯O5	0.84 (4)	1.97 (2)	2.735 (2)	150 (3)
O3*W*—H3*WA*⋯O1^vi^	0.84 (2)	1.95 (2)	2.780 (2)	176 (4)
O2*W*—H2*WB*⋯N2^i^	0.83 (2)	2.02 (2)	2.835 (3)	166 (4)
O3—H3*B*⋯O12^v^	0.82	2.05	2.735 (3)	141
O3*W*—H3*WB*⋯O3	0.83 (4)	2.00 (4)	2.803 (3)	164 (4)

Symmetry codes: (i) 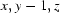; (ii) 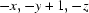; (iii) 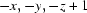; (iv) 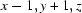; (v) 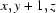; (vi) 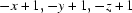.

**Fig. 1.**
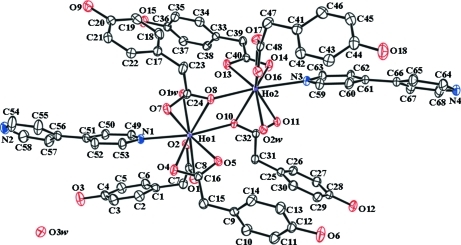
The molecular structure of the title complex, showing the atom–labelling scheme. Displacement ellipsoids are drawn at the 30% probability level. All hydrogen atoms are omitted for clarity.

## supplementary crystallographic information

### Comment

The design and synthesis of carboxylic mental–organic complexes have been an increasing interest for decades owing to their potential practical applications, such as fluorescence, magnetism (Wang, *et al.*, 2010; Fang, *et al.*, 2006; Wang, *et al.*, 2008). We have worked at it before (Liu, *et al.*, 2010). Herein we report the crystal structure of a new holmium (III) complex with the ligand 4–hydroxyphenylacetate and 4,4'–bipyridine.

In the title dinuclear complex (I), the unique HoIII ion is coordinated by four HPAA ligands, a bipy ligand and a water molecule *via* eight O atoms and one N atom (see, Fig. 1). The HoIII ion is in a distorted tricapped trigonal prismatic environment. The HPAA ligands coordinate in the bis–chelate, bridging and bridging tridentate modes.

### Experimental

All reagents and solvents used were of commercially available quality and without purified before using. *p*-hydroxyphenylacetic acid(HPAA) (0.456 g, 3 mmol)and sodium hydroxide (0.12 g, 3 mmol) were mixed together in water(10 ml), then Ho[(NO_3_)_3_](0.351 g, 1 mmol) dissolved in water(10 ml) was added into the above solution, after stirred for an hour, an ethanol(5 ml) solution of 4,4'-bipyridine(0.156 g, 1 mmol) is slowly dripped into the above solution with stirring for three hours. After filtration, the filtrate was allowed to stand at room temperature, and single crystals suitable for *X*–ray work were obtained after a week.

### Refinement

All H atoms attached to C atoms and *O*(hydroxyl) atom were fixed geometrically and treated as riding with C—H = 0.97 Å (methylene) or 0.93 Å (aromatic) and O—H = 0.82 Å with *U*_iso_(H) = 1.2*U*_eq_(C) or *U*_iso_(H) = 1.5*U*_eq_(O). H atoms of water molecule were located in a difference Fourier map and included in the subsequent refinement using restraints (O—H= 0.82 (1)Å and H···H= 1.39 (2) Å) with *U*_iso_(H) = 1.5*U*_eq_(O). In the last cycles of refinement they were treated as riding on their parent O atom.

### Crystal data

**Table:**

[Ho_2_(C_8_H_7_O_3_)_6_(C_10_H_8_N_2_)_2_(H_2_O)_2_]·H_2_O	*Z* = 2
*M**_r_* = 1603.09	*F*(000) = 1604
Triclinic, *P*1	*D*_x_ = 1.689 Mg m^−^^3^
Hall symbol: -P 1	Mo *K*α radiation, λ = 0.71073 Å
*a* = 11.7217 (7) Å	Cell parameters from 9517 reflections
*b* = 16.1885 (9) Å	θ = 1.2–27.7°
*c* = 18.413 (1) Å	µ = 2.58 mm^−^^1^
α = 83.441 (3)°	*T* = 296 K
β = 72.208 (3)°	Block, colourless
γ = 71.337 (3)°	0.34 × 0.11 × 0.03 mm
*V* = 3151.4 (3) Å^3^	

### Data collection

**Table:**

Bruker APEXII CCD diffractometer	14593 independent reflections
Radiation source: fine-focus sealed tube	12826 reflections with *I* > 2σ(*I*)
graphite	*R*_int_ = 0.021
Detector resolution: 10.0 pixels mm^-1^	θ_max_ = 27.7°, θ_min_ = 1.8°
φ and ω scans	*h* = −15→15
Absorption correction: multi-scan (*SADABS*; Sheldrick, 1996)	*k* = −21→21
*T*_min_ = 0.469, *T*_max_ = 0.924	*l* = −23→24
49489 measured reflections	

### Refinement

**Table:**

Refinement on *F*^2^	Primary atom site location: structure-invariant direct methods
Least-squares matrix: full	Secondary atom site location: difference Fourier map
*R*[*F*^2^ > 2σ(*F*^2^)] = 0.019	Hydrogen site location: difference Fourier map
*wR*(*F*^2^) = 0.051	H atoms treated by a mixture of independent and constrained refinement
*S* = 1.00	*w* = 1/[σ^2^(*F*_o_^2^) + (0.0275*P*)^2^ + 1.0527*P*] where *P* = (*F*_o_^2^ + 2*F*_c_^2^)/3
14593 reflections	(Δ/σ)_max_ = 0.001
874 parameters	Δρ_max_ = 0.58 e Å^−^^3^
9 restraints	Δρ_min_ = −0.66 e Å^−^^3^

### Special details

**Table:**

Geometry. All e.s.d.'s (except the e.s.d. in the dihedral angle between two l.s. planes) are estimated using the full covariance matrix. The cell e.s.d.'s are taken into account individually in the estimation of e.s.d.'s in distances, angles and torsion angles; correlations between e.s.d.'s in cell parameters are only used when they are defined by crystal symmetry. An approximate (isotropic) treatment of cell e.s.d.'s is used for estimating e.s.d.'s involving l.s. planes.
Refinement. Refinement of *F*^2^ against ALL reflections. The weighted *R*-factor *wR* and goodness of fit *S* are based on *F*^2^, conventional *R*-factors *R* are based on *F*, with *F* set to zero for negative *F*^2^. The threshold expression of *F*^2^ > σ(*F*^2^) is used only for calculating *R*-factors(gt) *etc*. and is not relevant to the choice of reflections for refinement. *R*-factors based on *F*^2^ are statistically about twice as large as those based on *F*, and *R*- factors based on ALL data will be even larger.

### Fractional atomic coordinates and isotropic or equivalent isotropic displacement parameters (Å^2^)

**Table:**

	*x*	*y*	*z*	*U*_iso_*/*U*_eq_	
Ho1	0.272400 (8)	0.363861 (5)	0.282352 (5)	0.02378 (3)	
Ho2	0.131189 (8)	0.207796 (5)	0.197587 (5)	0.02360 (3)	
C1	0.2255 (2)	0.50833 (14)	0.51575 (12)	0.0339 (4)	
C2	0.3416 (2)	0.51407 (16)	0.47192 (13)	0.0428 (5)	
H2A	0.3981	0.4670	0.4425	0.051*	
C3	0.3755 (2)	0.58874 (17)	0.47101 (15)	0.0469 (6)	
H3A	0.4539	0.5917	0.4408	0.056*	
C4	0.2935 (2)	0.65838 (15)	0.51477 (14)	0.0418 (5)	
C5	0.1786 (2)	0.65301 (16)	0.56028 (15)	0.0473 (6)	
H5A	0.1237	0.6993	0.5912	0.057*	
C6	0.1449 (2)	0.57896 (15)	0.56007 (14)	0.0424 (5)	
H6A	0.0664	0.5764	0.5903	0.051*	
C7	0.1867 (2)	0.42691 (15)	0.51853 (12)	0.0410 (5)	
H7A	0.2322	0.3815	0.5474	0.049*	
H7B	0.0977	0.4395	0.5445	0.049*	
C8	0.2134 (2)	0.39506 (14)	0.43950 (12)	0.0339 (5)	
C9	0.6227 (2)	0.09004 (14)	0.30166 (14)	0.0370 (5)	
C10	0.6033 (3)	0.05890 (15)	0.37580 (15)	0.0502 (6)	
H10A	0.5898	0.0964	0.4144	0.060*	
C11	0.6034 (3)	−0.02635 (16)	0.39484 (14)	0.0484 (6)	
H11A	0.5909	−0.0459	0.4453	0.058*	
C12	0.6221 (2)	−0.08165 (14)	0.33832 (13)	0.0374 (5)	
C13	0.6396 (2)	−0.05163 (15)	0.26397 (14)	0.0442 (6)	
H13A	0.6516	−0.0889	0.2256	0.053*	
C14	0.6395 (2)	0.03307 (15)	0.24624 (14)	0.0436 (5)	
H14A	0.6509	0.0525	0.1958	0.052*	
C15	0.6247 (2)	0.18212 (14)	0.28123 (17)	0.0483 (6)	
H15A	0.6901	0.1817	0.2337	0.058*	
H15B	0.6474	0.2034	0.3204	0.058*	
C16	0.50330 (19)	0.24547 (13)	0.27237 (12)	0.0319 (4)	
C17	0.2730 (2)	0.45389 (15)	0.01323 (12)	0.0401 (5)	
C18	0.1469 (3)	0.50135 (17)	0.02833 (13)	0.0467 (6)	
H18A	0.0865	0.4747	0.0552	0.056*	
C19	0.1085 (2)	0.58773 (17)	0.00444 (13)	0.0445 (6)	
H19A	0.0234	0.6183	0.0149	0.053*	
C20	0.1983 (2)	0.62779 (14)	−0.03524 (13)	0.0380 (5)	
C21	0.3243 (2)	0.58181 (15)	−0.04995 (13)	0.0394 (5)	
H21A	0.3848	0.6087	−0.0761	0.047*	
C22	0.3604 (2)	0.49612 (15)	−0.02592 (13)	0.0414 (5)	
H22A	0.4456	0.4659	−0.0362	0.050*	
C23	0.3127 (3)	0.36046 (16)	0.04020 (14)	0.0552 (7)	
H23A	0.3977	0.3319	0.0101	0.066*	
H23B	0.2581	0.3305	0.0321	0.066*	
C24	0.3076 (2)	0.35309 (13)	0.12357 (12)	0.0312 (4)	
C25	0.1420 (2)	0.11440 (14)	0.47623 (12)	0.0364 (5)	
C26	0.0396 (2)	0.10703 (16)	0.53588 (13)	0.0410 (5)	
H26A	−0.0290	0.1562	0.5516	0.049*	
C27	0.0377 (2)	0.02730 (15)	0.57260 (13)	0.0412 (5)	
H27A	−0.0314	0.0233	0.6128	0.049*	
C28	0.1387 (2)	−0.04566 (14)	0.54911 (12)	0.0349 (5)	
C29	0.2423 (2)	−0.03932 (15)	0.49013 (13)	0.0404 (5)	
H29A	0.3110	−0.0885	0.4745	0.048*	
C30	0.2432 (2)	0.04038 (16)	0.45452 (13)	0.0418 (5)	
H30A	0.3133	0.0444	0.4151	0.050*	
C31	0.1441 (3)	0.19961 (15)	0.43403 (13)	0.0431 (6)	
H31A	0.2251	0.2079	0.4275	0.052*	
H31B	0.0805	0.2466	0.4653	0.052*	
C32	0.12173 (19)	0.20651 (13)	0.35688 (11)	0.0290 (4)	
C33	−0.25364 (18)	0.47954 (12)	0.24487 (12)	0.0298 (4)	
C34	−0.2108 (2)	0.51790 (14)	0.17465 (13)	0.0368 (5)	
H34A	−0.1722	0.4834	0.1316	0.044*	
C35	−0.2240 (2)	0.60572 (15)	0.16724 (13)	0.0400 (5)	
H35A	−0.1957	0.6301	0.1195	0.048*	
C36	−0.2793 (2)	0.65763 (13)	0.23065 (13)	0.0361 (5)	
C37	−0.3218 (2)	0.62081 (14)	0.30095 (13)	0.0400 (5)	
H37A	−0.3594	0.6554	0.3439	0.048*	
C38	−0.3087 (2)	0.53248 (14)	0.30778 (12)	0.0363 (5)	
H38A	−0.3376	0.5083	0.3555	0.044*	
C39	−0.24065 (19)	0.38357 (13)	0.25160 (14)	0.0354 (5)	
H39A	−0.2858	0.3709	0.3032	0.043*	
H39B	−0.2810	0.3707	0.2174	0.043*	
C40	−0.10807 (19)	0.32350 (12)	0.23410 (11)	0.0288 (4)	
C41	0.2337 (2)	0.05597 (15)	−0.04467 (12)	0.0389 (5)	
C42	0.3525 (2)	0.00523 (15)	−0.04098 (13)	0.0409 (5)	
H42A	0.4086	0.0325	−0.0363	0.049*	
C43	0.3894 (2)	−0.08454 (16)	−0.04406 (13)	0.0441 (5)	
H43A	0.4698	−0.1171	−0.0423	0.053*	
C44	0.3061 (2)	−0.12564 (16)	−0.04983 (14)	0.0453 (6)	
C45	0.1881 (2)	−0.07691 (17)	−0.05438 (14)	0.0482 (6)	
H45A	0.1322	−0.1045	−0.0589	0.058*	
C46	0.1529 (2)	0.01300 (17)	−0.05227 (14)	0.0448 (5)	
H46A	0.0735	0.0454	−0.0560	0.054*	
C47	0.1909 (3)	0.15430 (15)	−0.03824 (13)	0.0450 (6)	
H47A	0.2538	0.1778	−0.0736	0.054*	
H47B	0.1133	0.1784	−0.0523	0.054*	
C48	0.1701 (2)	0.18144 (13)	0.04186 (12)	0.0345 (5)	
C49	0.2293 (2)	0.57715 (14)	0.32192 (13)	0.0358 (5)	
H49A	0.1561	0.5700	0.3562	0.043*	
C50	0.2470 (2)	0.65866 (14)	0.31483 (13)	0.0376 (5)	
H50A	0.1867	0.7046	0.3441	0.045*	
C51	0.3548 (2)	0.67117 (13)	0.26400 (13)	0.0351 (5)	
C52	0.4423 (2)	0.59947 (14)	0.22431 (15)	0.0428 (5)	
H52A	0.5172	0.6045	0.1906	0.051*	
C53	0.4180 (2)	0.52035 (14)	0.23484 (14)	0.0391 (5)	
H53A	0.4785	0.4729	0.2077	0.047*	
C54	0.4497 (3)	0.8672 (2)	0.1707 (2)	0.0762 (10)	
H54A	0.4979	0.8816	0.1236	0.091*	
C55	0.4422 (3)	0.78299 (17)	0.18155 (19)	0.0616 (8)	
H55A	0.4833	0.7430	0.1424	0.074*	
C56	0.3727 (2)	0.75905 (14)	0.25123 (15)	0.0420 (5)	
C57	0.3171 (3)	0.82188 (17)	0.30642 (17)	0.0620 (8)	
H57A	0.2716	0.8089	0.3549	0.074*	
C58	0.3296 (4)	0.90415 (18)	0.2890 (2)	0.0717 (9)	
H58A	0.2901	0.9456	0.3270	0.086*	
C59	0.1743 (2)	−0.00090 (14)	0.14909 (13)	0.0374 (5)	
H59A	0.2479	0.0087	0.1175	0.045*	
C60	0.1602 (2)	−0.08289 (14)	0.15042 (14)	0.0427 (5)	
H60A	0.2228	−0.1263	0.1193	0.051*	
C61	0.0537 (2)	−0.10042 (13)	0.19765 (13)	0.0362 (5)	
C62	−0.0384 (2)	−0.03130 (14)	0.23854 (14)	0.0403 (5)	
H62A	−0.1136	−0.0389	0.2695	0.048*	
C63	−0.0188 (2)	0.04952 (14)	0.23344 (13)	0.0372 (5)	
H63A	−0.0826	0.0952	0.2610	0.045*	
C64	0.1143 (4)	−0.33931 (17)	0.16825 (19)	0.0686 (9)	
H64A	0.1689	−0.3818	0.1333	0.082*	
C65	0.1212 (3)	−0.25481 (16)	0.15414 (16)	0.0611 (8)	
H65A	0.1778	−0.2415	0.1102	0.073*	
C66	0.0430 (2)	−0.19037 (14)	0.20594 (15)	0.0431 (6)	
C67	−0.0414 (3)	−0.21480 (17)	0.2689 (2)	0.0596 (8)	
H67A	−0.0966	−0.1740	0.3052	0.072*	
C68	−0.0425 (3)	−0.30033 (18)	0.2768 (2)	0.0700 (10)	
H68A	−0.1011	−0.3151	0.3188	0.084*	
N1	0.31240 (16)	0.50831 (11)	0.28188 (10)	0.0318 (4)	
N2	0.3934 (2)	0.92852 (15)	0.22255 (18)	0.0665 (7)	
N3	0.08768 (17)	0.06482 (11)	0.19077 (10)	0.0319 (4)	
N4	0.0345 (3)	−0.36303 (14)	0.22865 (18)	0.0661 (8)	
O1W	0.08841 (15)	0.45768 (9)	0.25373 (10)	0.0375 (3)	
O1	0.31207 (16)	0.33509 (11)	0.41074 (9)	0.0449 (4)	
O2	0.13821 (14)	0.43028 (10)	0.40070 (9)	0.0390 (3)	
O2W	0.32317 (15)	0.11326 (10)	0.21614 (10)	0.0385 (4)	
O3W	0.55642 (19)	0.76700 (13)	0.49155 (11)	0.0557 (5)	
O3	0.32922 (18)	0.73204 (12)	0.51196 (12)	0.0642 (5)	
H3B	0.2866	0.7610	0.5501	0.077*	
O4	0.49390 (14)	0.32486 (9)	0.26302 (10)	0.0440 (4)	
O5	0.41123 (14)	0.21924 (9)	0.27591 (9)	0.0374 (3)	
O6	0.6248 (2)	−0.16737 (10)	0.35245 (10)	0.0585 (5)	
H6B	0.6007	−0.1759	0.3986	0.088*	
O7	0.35194 (16)	0.39916 (11)	0.14949 (9)	0.0432 (4)	
O8	0.25598 (13)	0.30054 (9)	0.16713 (8)	0.0296 (3)	
O9	0.16649 (17)	0.71289 (11)	−0.06020 (11)	0.0534 (4)	
H9A	0.0953	0.7271	−0.0649	0.080*	
O10	0.14435 (13)	0.26933 (9)	0.31272 (8)	0.0289 (3)	
O11	0.08307 (15)	0.15300 (10)	0.33486 (8)	0.0362 (3)	
O12	0.14155 (16)	−0.12656 (10)	0.58262 (9)	0.0455 (4)	
H12A	0.0698	−0.1270	0.6056	0.068*	
O13	−0.01418 (13)	0.35135 (9)	0.20625 (9)	0.0359 (3)	
O14	−0.09026 (14)	0.24277 (9)	0.24609 (10)	0.0405 (4)	
O15	−0.28940 (18)	0.74419 (10)	0.22026 (10)	0.0534 (5)	
H15C	−0.3157	0.7676	0.2619	0.080*	
O16	0.25825 (15)	0.15694 (10)	0.07199 (9)	0.0381 (3)	
O17	0.06326 (16)	0.22766 (11)	0.07975 (9)	0.0456 (4)	
O18	0.3461 (2)	−0.21492 (12)	−0.05103 (13)	0.0715 (6)	
H18B	0.2894	−0.2326	−0.0537	0.107*	
H1WA	0.039 (3)	0.438 (2)	0.241 (2)	0.107*	
H1WB	0.063 (3)	0.5107 (12)	0.244 (2)	0.107*	
H2WA	0.373 (3)	0.129 (2)	0.232 (2)	0.107*	
H3WA	0.595 (3)	0.734 (2)	0.5203 (19)	0.107*	
H2WB	0.356 (3)	0.0595 (11)	0.214 (2)	0.107*	
H3WB	0.498 (3)	0.747 (2)	0.495 (2)	0.107*	

### Atomic displacement parameters (Å^2^)

**Table:**

	*U*^11^	*U*^22^	*U*^33^	*U*^12^	*U*^13^	*U*^23^
Ho1	0.02687 (5)	0.01618 (4)	0.03159 (5)	−0.00914 (3)	−0.01130 (4)	0.00250 (3)
Ho2	0.02580 (5)	0.01518 (4)	0.03182 (5)	−0.00741 (3)	−0.01004 (4)	0.00031 (3)
C1	0.0373 (11)	0.0366 (11)	0.0311 (10)	−0.0135 (10)	−0.0114 (9)	−0.0026 (9)
C2	0.0382 (12)	0.0431 (13)	0.0429 (12)	−0.0099 (10)	−0.0031 (10)	−0.0148 (10)
C3	0.0343 (12)	0.0511 (15)	0.0522 (14)	−0.0162 (11)	−0.0017 (10)	−0.0081 (12)
C4	0.0383 (12)	0.0361 (12)	0.0549 (14)	−0.0141 (10)	−0.0150 (11)	−0.0031 (11)
C5	0.0394 (13)	0.0391 (13)	0.0581 (15)	−0.0078 (11)	−0.0068 (11)	−0.0117 (11)
C6	0.0333 (12)	0.0428 (13)	0.0484 (13)	−0.0131 (10)	−0.0032 (10)	−0.0091 (11)
C7	0.0540 (14)	0.0383 (12)	0.0331 (11)	−0.0198 (11)	−0.0098 (10)	−0.0006 (9)
C8	0.0441 (12)	0.0300 (11)	0.0335 (10)	−0.0218 (10)	−0.0094 (9)	0.0019 (9)
C9	0.0281 (11)	0.0256 (10)	0.0594 (14)	−0.0034 (8)	−0.0209 (10)	0.0004 (10)
C10	0.0704 (18)	0.0300 (12)	0.0538 (15)	−0.0072 (12)	−0.0272 (13)	−0.0113 (11)
C11	0.0695 (17)	0.0353 (12)	0.0393 (12)	−0.0118 (12)	−0.0177 (12)	−0.0016 (10)
C12	0.0458 (13)	0.0249 (10)	0.0414 (12)	−0.0100 (10)	−0.0129 (10)	−0.0008 (9)
C13	0.0620 (16)	0.0325 (12)	0.0394 (12)	−0.0143 (11)	−0.0141 (11)	−0.0063 (10)
C14	0.0530 (14)	0.0361 (12)	0.0398 (12)	−0.0124 (11)	−0.0136 (11)	0.0048 (10)
C15	0.0361 (12)	0.0277 (11)	0.0859 (19)	−0.0078 (10)	−0.0286 (13)	0.0058 (12)
C16	0.0310 (10)	0.0224 (9)	0.0424 (11)	−0.0068 (8)	−0.0125 (9)	0.0007 (8)
C17	0.0673 (16)	0.0367 (12)	0.0286 (10)	−0.0281 (12)	−0.0200 (10)	0.0052 (9)
C18	0.0623 (16)	0.0573 (16)	0.0330 (11)	−0.0399 (14)	−0.0100 (11)	0.0037 (11)
C19	0.0423 (13)	0.0542 (15)	0.0421 (13)	−0.0199 (12)	−0.0117 (10)	−0.0069 (11)
C20	0.0499 (13)	0.0334 (11)	0.0394 (12)	−0.0177 (10)	−0.0202 (10)	0.0011 (9)
C21	0.0417 (13)	0.0351 (12)	0.0477 (13)	−0.0201 (10)	−0.0151 (10)	0.0055 (10)
C22	0.0491 (14)	0.0366 (12)	0.0461 (13)	−0.0184 (11)	−0.0211 (11)	0.0064 (10)
C23	0.106 (2)	0.0356 (13)	0.0369 (12)	−0.0365 (15)	−0.0262 (14)	0.0072 (10)
C24	0.0393 (11)	0.0249 (10)	0.0319 (10)	−0.0140 (9)	−0.0108 (9)	0.0038 (8)
C25	0.0542 (14)	0.0332 (11)	0.0333 (11)	−0.0227 (11)	−0.0214 (10)	0.0074 (9)
C26	0.0502 (13)	0.0330 (11)	0.0400 (12)	−0.0118 (10)	−0.0149 (10)	0.0017 (9)
C27	0.0446 (13)	0.0405 (13)	0.0381 (12)	−0.0179 (11)	−0.0081 (10)	0.0060 (10)
C28	0.0441 (12)	0.0325 (11)	0.0356 (11)	−0.0192 (10)	−0.0173 (9)	0.0086 (9)
C29	0.0412 (13)	0.0364 (12)	0.0422 (12)	−0.0118 (10)	−0.0121 (10)	0.0053 (10)
C30	0.0464 (13)	0.0458 (14)	0.0380 (12)	−0.0239 (11)	−0.0122 (10)	0.0108 (10)
C31	0.0705 (17)	0.0348 (12)	0.0390 (12)	−0.0298 (12)	−0.0258 (11)	0.0090 (10)
C32	0.0303 (10)	0.0243 (10)	0.0341 (10)	−0.0115 (8)	−0.0095 (8)	0.0033 (8)
C33	0.0260 (10)	0.0218 (9)	0.0404 (11)	−0.0028 (8)	−0.0112 (8)	−0.0040 (8)
C34	0.0386 (12)	0.0324 (11)	0.0355 (11)	−0.0057 (9)	−0.0065 (9)	−0.0103 (9)
C35	0.0481 (13)	0.0340 (12)	0.0368 (11)	−0.0141 (10)	−0.0095 (10)	0.0014 (9)
C36	0.0413 (12)	0.0235 (10)	0.0462 (12)	−0.0080 (9)	−0.0172 (10)	−0.0026 (9)
C37	0.0492 (14)	0.0301 (11)	0.0379 (12)	−0.0057 (10)	−0.0119 (10)	−0.0099 (9)
C38	0.0384 (12)	0.0306 (11)	0.0341 (11)	−0.0056 (9)	−0.0073 (9)	−0.0006 (9)
C39	0.0285 (10)	0.0232 (10)	0.0523 (13)	−0.0057 (8)	−0.0097 (9)	−0.0029 (9)
C40	0.0307 (10)	0.0223 (9)	0.0354 (10)	−0.0070 (8)	−0.0122 (8)	−0.0037 (8)
C41	0.0539 (14)	0.0366 (12)	0.0273 (10)	−0.0167 (11)	−0.0093 (10)	−0.0024 (9)
C42	0.0458 (13)	0.0393 (12)	0.0407 (12)	−0.0190 (11)	−0.0103 (10)	−0.0011 (10)
C43	0.0457 (13)	0.0394 (13)	0.0467 (13)	−0.0131 (11)	−0.0126 (11)	0.0000 (11)
C44	0.0549 (15)	0.0354 (12)	0.0449 (13)	−0.0178 (11)	−0.0077 (11)	−0.0035 (10)
C45	0.0517 (15)	0.0485 (15)	0.0509 (14)	−0.0255 (13)	−0.0111 (12)	−0.0062 (12)
C46	0.0425 (13)	0.0472 (14)	0.0440 (13)	−0.0126 (11)	−0.0095 (10)	−0.0084 (11)
C47	0.0644 (16)	0.0331 (12)	0.0338 (11)	−0.0086 (11)	−0.0154 (11)	−0.0002 (10)
C48	0.0484 (13)	0.0204 (10)	0.0355 (11)	−0.0146 (9)	−0.0104 (10)	0.0043 (8)
C49	0.0376 (12)	0.0291 (11)	0.0421 (12)	−0.0134 (9)	−0.0104 (9)	0.0009 (9)
C50	0.0431 (13)	0.0227 (10)	0.0462 (12)	−0.0086 (9)	−0.0120 (10)	−0.0036 (9)
C51	0.0397 (12)	0.0235 (10)	0.0493 (12)	−0.0127 (9)	−0.0214 (10)	0.0036 (9)
C52	0.0332 (12)	0.0287 (11)	0.0658 (16)	−0.0139 (9)	−0.0098 (11)	0.0034 (11)
C53	0.0326 (11)	0.0242 (10)	0.0581 (14)	−0.0092 (9)	−0.0078 (10)	−0.0033 (10)
C54	0.0502 (17)	0.0416 (16)	0.125 (3)	−0.0214 (14)	−0.0102 (18)	0.0248 (18)
C55	0.0448 (15)	0.0337 (13)	0.092 (2)	−0.0139 (12)	0.0011 (14)	0.0046 (14)
C56	0.0424 (13)	0.0261 (11)	0.0664 (16)	−0.0150 (10)	−0.0261 (12)	0.0075 (10)
C57	0.100 (2)	0.0359 (14)	0.0655 (18)	−0.0316 (15)	−0.0346 (17)	0.0010 (12)
C58	0.114 (3)	0.0349 (14)	0.090 (2)	−0.0325 (17)	−0.053 (2)	0.0001 (15)
C59	0.0446 (13)	0.0261 (10)	0.0403 (12)	−0.0136 (10)	−0.0081 (10)	0.0017 (9)
C60	0.0568 (15)	0.0240 (10)	0.0462 (13)	−0.0092 (10)	−0.0153 (11)	−0.0028 (9)
C61	0.0507 (13)	0.0227 (10)	0.0475 (12)	−0.0154 (9)	−0.0288 (11)	0.0053 (9)
C62	0.0392 (12)	0.0278 (11)	0.0590 (14)	−0.0167 (10)	−0.0161 (11)	0.0045 (10)
C63	0.0378 (12)	0.0233 (10)	0.0518 (13)	−0.0108 (9)	−0.0121 (10)	−0.0028 (9)
C64	0.128 (3)	0.0303 (13)	0.073 (2)	−0.0278 (17)	−0.063 (2)	0.0027 (13)
C65	0.114 (3)	0.0322 (13)	0.0539 (15)	−0.0286 (15)	−0.0419 (17)	0.0030 (11)
C66	0.0579 (15)	0.0238 (10)	0.0644 (15)	−0.0176 (10)	−0.0391 (13)	0.0086 (10)
C67	0.0424 (14)	0.0329 (13)	0.106 (2)	−0.0159 (11)	−0.0238 (15)	0.0118 (14)
C68	0.0549 (17)	0.0355 (15)	0.132 (3)	−0.0245 (14)	−0.0416 (19)	0.0236 (17)
N1	0.0337 (9)	0.0210 (8)	0.0441 (10)	−0.0110 (7)	−0.0137 (8)	0.0009 (7)
N2	0.0637 (16)	0.0311 (12)	0.121 (2)	−0.0239 (12)	−0.0460 (16)	0.0181 (14)
N3	0.0396 (10)	0.0208 (8)	0.0386 (9)	−0.0114 (7)	−0.0142 (8)	0.0010 (7)
N4	0.0851 (18)	0.0308 (12)	0.114 (2)	−0.0268 (13)	−0.0706 (17)	0.0164 (13)
O1W	0.0416 (9)	0.0208 (7)	0.0570 (10)	−0.0086 (7)	−0.0259 (8)	0.0022 (7)
O1	0.0531 (10)	0.0380 (9)	0.0436 (9)	−0.0070 (8)	−0.0184 (8)	−0.0072 (7)
O2	0.0394 (9)	0.0426 (9)	0.0388 (8)	−0.0162 (7)	−0.0124 (7)	−0.0012 (7)
O2W	0.0376 (9)	0.0211 (7)	0.0629 (10)	−0.0066 (6)	−0.0259 (8)	0.0006 (7)
O3W	0.0592 (12)	0.0537 (12)	0.0591 (11)	−0.0238 (10)	−0.0228 (9)	0.0161 (9)
O3	0.0540 (11)	0.0463 (11)	0.0929 (15)	−0.0267 (9)	−0.0048 (10)	−0.0145 (10)
O4	0.0339 (8)	0.0229 (7)	0.0763 (12)	−0.0106 (6)	−0.0188 (8)	0.0097 (7)
O5	0.0325 (8)	0.0220 (7)	0.0630 (10)	−0.0063 (6)	−0.0219 (7)	−0.0052 (7)
O6	0.0981 (15)	0.0290 (9)	0.0480 (10)	−0.0264 (10)	−0.0128 (10)	−0.0004 (7)
O7	0.0595 (10)	0.0481 (10)	0.0366 (8)	−0.0382 (9)	−0.0137 (7)	0.0053 (7)
O8	0.0332 (7)	0.0205 (7)	0.0364 (7)	−0.0124 (6)	−0.0082 (6)	0.0023 (6)
O9	0.0584 (11)	0.0347 (9)	0.0772 (13)	−0.0157 (8)	−0.0348 (10)	0.0070 (9)
O10	0.0330 (7)	0.0225 (7)	0.0343 (7)	−0.0137 (6)	−0.0104 (6)	0.0052 (6)
O11	0.0507 (9)	0.0332 (8)	0.0361 (8)	−0.0270 (7)	−0.0161 (7)	0.0067 (6)
O12	0.0501 (10)	0.0329 (8)	0.0516 (10)	−0.0189 (8)	−0.0092 (8)	0.0106 (7)
O13	0.0278 (7)	0.0206 (7)	0.0606 (10)	−0.0065 (6)	−0.0140 (7)	−0.0045 (7)
O14	0.0321 (8)	0.0206 (7)	0.0634 (10)	−0.0071 (6)	−0.0083 (7)	0.0026 (7)
O15	0.0780 (13)	0.0254 (8)	0.0601 (11)	−0.0152 (8)	−0.0246 (10)	−0.0012 (8)
O16	0.0400 (9)	0.0354 (8)	0.0410 (8)	−0.0133 (7)	−0.0110 (7)	−0.0053 (7)
O17	0.0494 (10)	0.0407 (9)	0.0406 (9)	0.0021 (8)	−0.0185 (8)	−0.0066 (7)
O18	0.0770 (14)	0.0343 (10)	0.1083 (17)	−0.0191 (10)	−0.0299 (13)	−0.0047 (10)

### Geometric parameters (Å, °)

**Table:**

Ho1—O5	2.3771 (14)	C32—O11	1.251 (2)
Ho1—O1W	2.3772 (15)	C32—O10	1.272 (2)
Ho1—O10	2.3810 (12)	C33—C38	1.382 (3)
Ho1—O4	2.3882 (15)	C33—C34	1.389 (3)
Ho1—O2	2.3952 (15)	C33—C39	1.506 (3)
Ho1—O7	2.4101 (15)	C34—C35	1.375 (3)
Ho1—O1	2.5107 (16)	C34—H34A	0.9300
Ho1—N1	2.5264 (16)	C35—C36	1.379 (3)
Ho1—O8	2.5420 (14)	C35—H35A	0.9300
Ho1—C16	2.737 (2)	C36—O15	1.364 (2)
Ho1—C8	2.827 (2)	C36—C37	1.378 (3)
Ho1—C24	2.843 (2)	C37—C38	1.384 (3)
Ho2—O8	2.3281 (13)	C37—H37A	0.9300
Ho2—O14	2.3695 (15)	C38—H38A	0.9300
Ho2—O2W	2.3809 (15)	C39—C40	1.504 (3)
Ho2—O13	2.3903 (14)	C39—H39A	0.9700
Ho2—O16	2.4062 (15)	C39—H39B	0.9700
Ho2—O17	2.4855 (16)	C40—O13	1.264 (2)
Ho2—O10	2.5081 (14)	C40—O14	1.260 (2)
Ho2—O11	2.5359 (14)	C41—C42	1.390 (3)
Ho2—N3	2.5483 (16)	C41—C46	1.387 (3)
Ho2—C40	2.757 (2)	C41—C47	1.515 (3)
Ho2—C48	2.823 (2)	C42—C43	1.380 (3)
Ho2—C32	2.899 (2)	C42—H42A	0.9300
C1—C2	1.381 (3)	C43—C44	1.379 (3)
C1—C6	1.384 (3)	C43—H43A	0.9300
C1—C7	1.516 (3)	C44—O18	1.370 (3)
C2—C3	1.385 (3)	C44—C45	1.378 (4)
C2—H2A	0.9300	C45—C46	1.382 (3)
C3—C4	1.373 (3)	C45—H45A	0.9300
C3—H3A	0.9300	C46—H46A	0.9300
C4—O3	1.376 (3)	C47—C48	1.512 (3)
C4—C5	1.376 (3)	C47—H47A	0.9700
C5—C6	1.377 (3)	C47—H47B	0.9700
C5—H5A	0.9300	C48—O16	1.253 (3)
C6—H6A	0.9300	C48—O17	1.265 (3)
C7—C8	1.507 (3)	C49—N1	1.337 (3)
C7—H7A	0.9700	C49—C50	1.386 (3)
C7—H7B	0.9700	C49—H49A	0.9300
C8—O1	1.259 (3)	C50—C51	1.383 (3)
C8—O2	1.257 (3)	C50—H50A	0.9300
C9—C10	1.377 (3)	C51—C52	1.381 (3)
C9—C14	1.381 (3)	C51—C56	1.486 (3)
C9—C15	1.501 (3)	C52—C53	1.379 (3)
C10—C11	1.384 (3)	C52—H52A	0.9300
C10—H10A	0.9300	C53—N1	1.335 (3)
C11—C12	1.371 (3)	C53—H53A	0.9300
C11—H11A	0.9300	C54—N2	1.321 (4)
C12—O6	1.374 (2)	C54—C55	1.381 (4)
C12—C13	1.376 (3)	C54—H54A	0.9300
C13—C14	1.372 (3)	C55—C56	1.382 (4)
C13—H13A	0.9300	C55—H55A	0.9300
C14—H14A	0.9300	C56—C57	1.378 (4)
C15—C16	1.508 (3)	C57—C58	1.377 (4)
C15—H15A	0.9700	C57—H57A	0.9300
C15—H15B	0.9700	C58—N2	1.315 (4)
C16—O4	1.251 (2)	C58—H58A	0.9300
C16—O5	1.262 (2)	C59—N3	1.331 (3)
C17—C18	1.386 (4)	C59—C60	1.386 (3)
C17—C22	1.387 (3)	C59—H59A	0.9300
C17—C23	1.507 (3)	C60—C61	1.379 (3)
C18—C19	1.389 (4)	C60—H60A	0.9300
C18—H18A	0.9300	C61—C62	1.381 (3)
C19—C20	1.387 (3)	C61—C66	1.488 (3)
C19—H19A	0.9300	C62—C63	1.388 (3)
C20—O9	1.371 (3)	C62—H62A	0.9300
C20—C21	1.380 (3)	C63—N3	1.338 (3)
C21—C22	1.378 (3)	C63—H63A	0.9300
C21—H21A	0.9300	C64—N4	1.325 (4)
C22—H22A	0.9300	C64—C65	1.386 (3)
C23—C24	1.510 (3)	C64—H64A	0.9300
C23—H23A	0.9700	C65—C66	1.385 (4)
C23—H23B	0.9700	C65—H65A	0.9300
C24—O7	1.239 (2)	C66—C67	1.387 (4)
C24—O8	1.269 (2)	C67—C68	1.379 (3)
C25—C30	1.383 (3)	C67—H67A	0.9300
C25—C26	1.385 (3)	C68—N4	1.327 (4)
C25—C31	1.508 (3)	C68—H68A	0.9300
C26—C27	1.391 (3)	O1W—H1WA	0.84 (4)
C26—H26A	0.9300	O1W—H1WB	0.831 (17)
C27—C28	1.375 (3)	O2W—H2WA	0.84 (4)
C27—H27A	0.9300	O2W—H2WB	0.831 (17)
C28—O12	1.377 (2)	O3W—H3WA	0.837 (18)
C28—C29	1.383 (3)	O3W—H3WB	0.83 (4)
C29—C30	1.381 (3)	O3—H3B	0.8200
C29—H29A	0.9300	O6—H6B	0.8200
C30—H30A	0.9300	O9—H9A	0.8200
C31—C32	1.507 (3)	O12—H12A	0.8200
C31—H31A	0.9700	O15—H15C	0.8200
C31—H31B	0.9700	O18—H18B	0.8200
			
O5—Ho1—O1W	145.55 (5)	C22—C21—H21A	120.0
O5—Ho1—O10	73.53 (5)	C20—C21—H21A	120.0
O1W—Ho1—O10	79.75 (5)	C21—C22—C17	121.6 (2)
O5—Ho1—O4	54.41 (5)	C21—C22—H22A	119.2
O1W—Ho1—O4	149.28 (5)	C17—C22—H22A	119.2
O10—Ho1—O4	127.60 (5)	C17—C23—C24	112.30 (19)
O5—Ho1—O2	122.44 (6)	C17—C23—H23A	109.1
O1W—Ho1—O2	74.72 (6)	C24—C23—H23A	109.1
O10—Ho1—O2	83.89 (5)	C17—C23—H23B	109.1
O4—Ho1—O2	117.35 (6)	C24—C23—H23B	109.1
O5—Ho1—O7	95.36 (6)	H23A—C23—H23B	107.9
O1W—Ho1—O7	77.82 (6)	O7—C24—O8	120.20 (19)
O10—Ho1—O7	117.25 (5)	O7—C24—C23	120.08 (18)
O4—Ho1—O7	76.66 (6)	O8—C24—C23	119.71 (19)
O2—Ho1—O7	141.40 (6)	O7—C24—Ho1	57.27 (10)
O5—Ho1—O1	75.36 (5)	O8—C24—Ho1	63.40 (10)
O1W—Ho1—O1	127.14 (6)	C23—C24—Ho1	171.67 (17)
O10—Ho1—O1	91.10 (5)	C30—C25—C26	118.2 (2)
O4—Ho1—O1	72.00 (6)	C30—C25—C31	119.9 (2)
O2—Ho1—O1	52.49 (5)	C26—C25—C31	121.9 (2)
O7—Ho1—O1	146.74 (6)	C25—C26—C27	121.0 (2)
O5—Ho1—N1	130.43 (5)	C25—C26—H26A	119.5
O1W—Ho1—N1	80.93 (5)	C27—C26—H26A	119.5
O10—Ho1—N1	153.96 (5)	C28—C27—C26	119.6 (2)
O4—Ho1—N1	76.19 (5)	C28—C27—H27A	120.2
O2—Ho1—N1	74.28 (5)	C26—C27—H27A	120.2
O7—Ho1—N1	75.05 (5)	O12—C28—C27	122.3 (2)
O1—Ho1—N1	86.78 (6)	O12—C28—C29	117.6 (2)
O5—Ho1—O8	75.80 (5)	C27—C28—C29	120.1 (2)
O1W—Ho1—O8	73.53 (5)	C30—C29—C28	119.7 (2)
O10—Ho1—O8	65.56 (4)	C30—C29—H29A	120.2
O4—Ho1—O8	103.12 (5)	C28—C29—H29A	120.2
O2—Ho1—O8	139.03 (5)	C29—C30—C25	121.3 (2)
O7—Ho1—O8	52.00 (4)	C29—C30—H30A	119.3
O1—Ho1—O8	147.05 (5)	C25—C30—H30A	119.3
N1—Ho1—O8	124.48 (5)	C25—C31—C32	114.81 (17)
O5—Ho1—C16	27.44 (5)	C25—C31—H31A	108.6
O1W—Ho1—C16	163.71 (6)	C32—C31—H31A	108.6
O10—Ho1—C16	100.44 (5)	C25—C31—H31B	108.6
O4—Ho1—C16	27.17 (5)	C32—C31—H31B	108.6
O2—Ho1—C16	121.57 (6)	H31A—C31—H31B	107.5
O7—Ho1—C16	87.84 (6)	O11—C32—O10	119.33 (18)
O1—Ho1—C16	69.11 (6)	O11—C32—C31	122.74 (17)
N1—Ho1—C16	102.99 (6)	O10—C32—C31	117.93 (17)
O8—Ho1—C16	91.60 (5)	O11—C32—Ho2	60.80 (10)
O5—Ho1—C8	100.17 (6)	O10—C32—Ho2	59.60 (10)
O1W—Ho1—C8	100.93 (6)	C31—C32—Ho2	168.90 (16)
O10—Ho1—C8	89.48 (5)	C38—C33—C34	117.70 (19)
O4—Ho1—C8	93.78 (6)	C38—C33—C39	121.50 (19)
O2—Ho1—C8	26.23 (6)	C34—C33—C39	120.79 (19)
O7—Ho1—C8	152.11 (5)	C35—C34—C33	121.6 (2)
O1—Ho1—C8	26.45 (6)	C35—C34—H34A	119.2
N1—Ho1—C8	77.24 (6)	C33—C34—H34A	119.2
O8—Ho1—C8	154.94 (5)	C34—C35—C36	120.0 (2)
C16—Ho1—C8	95.37 (7)	C34—C35—H35A	120.0
O5—Ho1—C24	87.10 (6)	C36—C35—H35A	120.0
O1W—Ho1—C24	72.16 (6)	O15—C36—C37	122.9 (2)
O10—Ho1—C24	91.64 (5)	O15—C36—C35	117.6 (2)
O4—Ho1—C24	91.29 (6)	C37—C36—C35	119.4 (2)
O2—Ho1—C24	146.84 (6)	C36—C37—C38	120.2 (2)
O7—Ho1—C24	25.63 (5)	C36—C37—H37A	119.9
O1—Ho1—C24	160.67 (6)	C38—C37—H37A	119.9
N1—Ho1—C24	98.87 (6)	C37—C38—C33	121.1 (2)
O8—Ho1—C24	26.51 (5)	C37—C38—H38A	119.4
C16—Ho1—C24	91.57 (6)	C33—C38—H38A	119.4
C8—Ho1—C24	172.65 (6)	C40—C39—C33	115.48 (17)
O8—Ho2—O14	128.97 (5)	C40—C39—H39A	108.4
O8—Ho2—O2W	78.65 (5)	C33—C39—H39A	108.4
O14—Ho2—O2W	143.15 (6)	C40—C39—H39B	108.4
O8—Ho2—O13	75.24 (5)	C33—C39—H39B	108.4
O14—Ho2—O13	54.39 (5)	H39A—C39—H39B	107.5
O2W—Ho2—O13	148.14 (5)	O13—C40—O14	119.05 (18)
O8—Ho2—O16	80.37 (5)	O13—C40—C39	122.00 (17)
O14—Ho2—O16	127.07 (5)	O14—C40—C39	118.94 (17)
O2W—Ho2—O16	75.48 (6)	O13—C40—Ho2	60.01 (10)
O13—Ho2—O16	117.05 (5)	O14—C40—Ho2	59.05 (10)
O8—Ho2—O17	99.18 (5)	C39—C40—Ho2	176.83 (14)
O14—Ho2—O17	77.29 (6)	C42—C41—C46	117.5 (2)
O2W—Ho2—O17	127.31 (6)	C42—C41—C47	122.0 (2)
O13—Ho2—O17	75.26 (5)	C46—C41—C47	120.5 (2)
O16—Ho2—O17	52.69 (5)	C43—C42—C41	121.8 (2)
O8—Ho2—O10	66.87 (5)	C43—C42—H42A	119.1
O14—Ho2—O10	91.38 (5)	C41—C42—H42A	119.1
O2W—Ho2—O10	77.13 (5)	C44—C43—C42	119.5 (2)
O13—Ho2—O10	76.04 (5)	C44—C43—H43A	120.2
O16—Ho2—O10	140.63 (5)	C42—C43—H43A	120.2
O17—Ho2—O10	150.53 (5)	O18—C44—C43	117.4 (2)
O8—Ho2—O11	115.52 (5)	O18—C44—C45	122.7 (2)
O14—Ho2—O11	72.89 (5)	C43—C44—C45	119.9 (2)
O2W—Ho2—O11	72.74 (6)	C46—C45—C44	120.0 (2)
O13—Ho2—O11	102.59 (5)	C46—C45—H45A	120.0
O16—Ho2—O11	140.14 (5)	C44—C45—H45A	120.0
O17—Ho2—O11	143.72 (5)	C45—C46—C41	121.3 (2)
O10—Ho2—O11	51.14 (4)	C45—C46—H46A	119.3
O8—Ho2—N3	153.75 (5)	C41—C46—H46A	119.3
O14—Ho2—N3	76.13 (5)	C48—C47—C41	111.53 (18)
O2W—Ho2—N3	83.12 (5)	C48—C47—H47A	109.3
O13—Ho2—N3	127.19 (5)	C41—C47—H47A	109.3
O16—Ho2—N3	76.86 (5)	C48—C47—H47B	109.3
O17—Ho2—N3	77.14 (6)	C41—C47—H47B	109.3
O10—Ho2—N3	126.91 (5)	H47A—C47—H47B	108.0
O11—Ho2—N3	76.05 (5)	O16—C48—O17	119.2 (2)
O8—Ho2—C40	102.25 (5)	O16—C48—C47	120.2 (2)
O14—Ho2—C40	27.14 (5)	O17—C48—C47	120.6 (2)
O2W—Ho2—C40	158.35 (6)	O16—C48—Ho2	58.01 (11)
O13—Ho2—C40	27.26 (5)	O17—C48—Ho2	61.66 (11)
O16—Ho2—C40	126.15 (6)	C47—C48—Ho2	171.92 (14)
O17—Ho2—C40	74.19 (6)	N1—C49—C50	123.2 (2)
O10—Ho2—C40	83.34 (5)	N1—C49—H49A	118.4
O11—Ho2—C40	87.80 (6)	C50—C49—H49A	118.4
N3—Ho2—C40	101.66 (6)	C51—C50—C49	119.5 (2)
O8—Ho2—C48	91.48 (5)	C51—C50—H50A	120.2
O14—Ho2—C48	101.90 (6)	C49—C50—H50A	120.2
O2W—Ho2—C48	100.89 (6)	C50—C51—C52	117.22 (19)
O13—Ho2—C48	97.73 (6)	C50—C51—C56	120.9 (2)
O16—Ho2—C48	26.21 (6)	C52—C51—C56	121.9 (2)
O17—Ho2—C48	26.61 (6)	C53—C52—C51	119.7 (2)
O10—Ho2—C48	158.30 (5)	C53—C52—H52A	120.1
O11—Ho2—C48	149.46 (5)	C51—C52—H52A	120.1
N3—Ho2—C48	73.52 (6)	N1—C53—C52	123.5 (2)
C40—Ho2—C48	100.70 (6)	N1—C53—H53A	118.3
O8—Ho2—C32	90.57 (5)	C52—C53—H53A	118.3
O14—Ho2—C32	83.95 (6)	N2—C54—C55	124.6 (3)
O2W—Ho2—C32	70.40 (6)	N2—C54—H54A	117.7
O13—Ho2—C32	91.80 (6)	C55—C54—H54A	117.7
O16—Ho2—C32	145.79 (6)	C56—C55—C54	119.1 (3)
O17—Ho2—C32	161.07 (6)	C56—C55—H55A	120.4
O10—Ho2—C32	25.94 (5)	C54—C55—H55A	120.4
O11—Ho2—C32	25.50 (5)	C57—C56—C55	116.6 (2)
N3—Ho2—C32	100.97 (5)	C57—C56—C51	122.3 (2)
C40—Ho2—C32	87.95 (6)	C55—C56—C51	121.1 (2)
C48—Ho2—C32	170.46 (6)	C56—C57—C58	119.3 (3)
C2—C1—C6	117.8 (2)	C56—C57—H57A	120.3
C2—C1—C7	122.2 (2)	C58—C57—H57A	120.3
C6—C1—C7	120.0 (2)	N2—C58—C57	124.9 (3)
C3—C2—C1	121.1 (2)	N2—C58—H58A	117.6
C3—C2—H2A	119.4	C57—C58—H58A	117.6
C1—C2—H2A	119.4	N3—C59—C60	123.2 (2)
C4—C3—C2	120.0 (2)	N3—C59—H59A	118.4
C4—C3—H3A	120.0	C60—C59—H59A	118.4
C2—C3—H3A	120.0	C61—C60—C59	120.3 (2)
O3—C4—C5	121.7 (2)	C61—C60—H60A	119.9
O3—C4—C3	118.6 (2)	C59—C60—H60A	119.9
C5—C4—C3	119.7 (2)	C60—C61—C62	116.46 (19)
C6—C5—C4	119.9 (2)	C60—C61—C66	120.9 (2)
C6—C5—H5A	120.0	C62—C61—C66	122.6 (2)
C4—C5—H5A	120.0	C61—C62—C63	120.1 (2)
C5—C6—C1	121.4 (2)	C61—C62—H62A	120.0
C5—C6—H6A	119.3	C63—C62—H62A	120.0
C1—C6—H6A	119.3	N3—C63—C62	123.1 (2)
C8—C7—C1	111.29 (17)	N3—C63—H63A	118.4
C8—C7—H7A	109.4	C62—C63—H63A	118.4
C1—C7—H7A	109.4	N4—C64—C65	123.8 (3)
C8—C7—H7B	109.4	N4—C64—H64A	118.1
C1—C7—H7B	109.4	C65—C64—H64A	118.1
H7A—C7—H7B	108.0	C66—C65—C64	119.5 (3)
O1—C8—O2	119.3 (2)	C66—C65—H65A	120.2
O1—C8—C7	120.8 (2)	C64—C65—H65A	120.2
O2—C8—C7	119.9 (2)	C67—C66—C65	116.8 (2)
O1—C8—Ho1	62.62 (11)	C67—C66—C61	121.0 (2)
O2—C8—Ho1	57.34 (11)	C65—C66—C61	122.1 (2)
C7—C8—Ho1	169.74 (14)	C68—C67—C66	119.1 (3)
C10—C9—C14	117.2 (2)	C68—C67—H67A	120.4
C10—C9—C15	121.9 (2)	C66—C67—H67A	120.4
C14—C9—C15	120.9 (2)	N4—C68—C67	124.5 (3)
C9—C10—C11	122.2 (2)	N4—C68—H68A	117.8
C9—C10—H10A	118.9	C67—C68—H68A	117.8
C11—C10—H10A	118.9	C53—N1—C49	116.75 (18)
C12—C11—C10	119.2 (2)	C53—N1—Ho1	119.11 (14)
C12—C11—H11A	120.4	C49—N1—Ho1	123.92 (13)
C10—C11—H11A	120.4	C54—N2—C58	115.4 (2)
O6—C12—C13	117.6 (2)	C63—N3—C59	116.68 (18)
O6—C12—C11	122.7 (2)	C63—N3—Ho2	121.80 (14)
C13—C12—C11	119.7 (2)	C59—N3—Ho2	121.30 (14)
C14—C13—C12	120.2 (2)	C68—N4—C64	116.2 (2)
C14—C13—H13A	119.9	Ho1—O1W—H1WA	122 (2)
C12—C13—H13A	119.9	Ho1—O1W—H1WB	135 (3)
C13—C14—C9	121.5 (2)	H1WA—O1W—H1WB	102 (2)
C13—C14—H14A	119.3	C8—O1—Ho1	90.93 (13)
C9—C14—H14A	119.3	C8—O2—Ho1	96.43 (13)
C9—C15—C16	115.55 (18)	Ho2—O2W—H2WA	125 (2)
C9—C15—H15A	108.4	Ho2—O2W—H2WB	134 (3)
C16—C15—H15A	108.4	H2WA—O2W—H2WB	101 (2)
C9—C15—H15B	108.4	H3WA—O3W—H3WB	103 (2)
C16—C15—H15B	108.4	C4—O3—H3B	109.5
H15A—C15—H15B	107.5	C16—O4—Ho1	92.12 (12)
O4—C16—O5	120.21 (19)	C16—O5—Ho1	92.35 (12)
O4—C16—C15	119.12 (18)	C12—O6—H6B	109.5
O5—C16—C15	120.65 (18)	C24—O7—Ho1	97.10 (12)
O4—C16—Ho1	60.70 (11)	C24—O8—Ho2	153.63 (13)
O5—C16—Ho1	60.21 (10)	C24—O8—Ho1	90.09 (11)
C15—C16—Ho1	170.30 (17)	Ho2—O8—Ho1	114.10 (5)
C18—C17—C22	117.6 (2)	C20—O9—H9A	109.5
C18—C17—C23	120.9 (2)	C32—O10—Ho1	142.47 (13)
C22—C17—C23	121.5 (2)	C32—O10—Ho2	94.46 (11)
C17—C18—C19	121.7 (2)	Ho1—O10—Ho2	113.46 (5)
C17—C18—H18A	119.1	C32—O11—Ho2	93.71 (11)
C19—C18—H18A	119.1	C28—O12—H12A	109.5
C20—C19—C18	119.3 (2)	C40—O13—Ho2	92.73 (11)
C20—C19—H19A	120.4	C40—O14—Ho2	93.81 (12)
C18—C19—H19A	120.4	C36—O15—H15C	109.5
O9—C20—C21	118.1 (2)	C48—O16—Ho2	95.78 (13)
O9—C20—C19	122.1 (2)	C48—O17—Ho2	91.73 (13)
C21—C20—C19	119.8 (2)	C44—O18—H18B	109.5
C22—C21—C20	120.0 (2)		
			
C6—C1—C2—C3	−1.4 (4)	O8—Ho1—N1—C53	−60.09 (18)
C7—C1—C2—C3	−179.2 (2)	C16—Ho1—N1—C53	41.04 (17)
C1—C2—C3—C4	0.6 (4)	C8—Ho1—N1—C53	133.69 (17)
C2—C3—C4—O3	−179.2 (2)	C24—Ho1—N1—C53	−52.65 (17)
C2—C3—C4—C5	1.0 (4)	O5—Ho1—N1—C49	−144.34 (16)
O3—C4—C5—C6	178.3 (2)	O1W—Ho1—N1—C49	51.71 (17)
C3—C4—C5—C6	−1.9 (4)	O10—Ho1—N1—C49	9.2 (2)
C4—C5—C6—C1	1.2 (4)	O4—Ho1—N1—C49	−148.96 (18)
C2—C1—C6—C5	0.5 (4)	O2—Ho1—N1—C49	−24.86 (16)
C7—C1—C6—C5	178.4 (2)	O7—Ho1—N1—C49	131.43 (17)
C2—C1—C7—C8	−48.5 (3)	O1—Ho1—N1—C49	−76.71 (17)
C6—C1—C7—C8	133.7 (2)	O8—Ho1—N1—C49	114.45 (16)
C1—C7—C8—O1	97.2 (2)	C16—Ho1—N1—C49	−144.41 (17)
C1—C7—C8—O2	−80.7 (3)	C8—Ho1—N1—C49	−51.77 (17)
C1—C7—C8—Ho1	−9.5 (11)	C24—Ho1—N1—C49	121.89 (17)
O5—Ho1—C8—O1	20.37 (13)	C55—C54—N2—C58	2.1 (5)
O1W—Ho1—C8—O1	172.98 (12)	C57—C58—N2—C54	−1.2 (5)
O10—Ho1—C8—O1	93.53 (12)	C62—C63—N3—C59	3.4 (3)
O4—Ho1—C8—O1	−34.12 (12)	C62—C63—N3—Ho2	−171.33 (17)
O2—Ho1—C8—O1	170.7 (2)	C60—C59—N3—C63	−2.3 (3)
O7—Ho1—C8—O1	−102.44 (17)	C60—C59—N3—Ho2	172.46 (17)
N1—Ho1—C8—O1	−109.04 (13)	O8—Ho2—N3—C63	174.44 (14)
O8—Ho1—C8—O1	98.58 (17)	O14—Ho2—N3—C63	−21.03 (16)
C16—Ho1—C8—O1	−6.91 (13)	O2W—Ho2—N3—C63	128.30 (17)
O5—Ho1—C8—O2	−150.31 (12)	O13—Ho2—N3—C63	−40.93 (18)
O1W—Ho1—C8—O2	2.30 (12)	O16—Ho2—N3—C63	−155.07 (17)
O10—Ho1—C8—O2	−77.15 (12)	O17—Ho2—N3—C63	−100.88 (17)
O4—Ho1—C8—O2	155.20 (12)	O10—Ho2—N3—C63	60.09 (18)
O7—Ho1—C8—O2	86.88 (17)	O11—Ho2—N3—C63	54.44 (16)
O1—Ho1—C8—O2	−170.7 (2)	C40—Ho2—N3—C63	−30.30 (17)
N1—Ho1—C8—O2	80.27 (12)	C48—Ho2—N3—C63	−128.16 (17)
O8—Ho1—C8—O2	−72.11 (18)	C32—Ho2—N3—C63	59.86 (17)
C16—Ho1—C8—O2	−177.59 (12)	O8—Ho2—N3—C59	0.0 (2)
O5—Ho1—C8—C7	132.4 (9)	O14—Ho2—N3—C59	164.50 (17)
O1W—Ho1—C8—C7	−75.0 (10)	O2W—Ho2—N3—C59	−46.18 (16)
O10—Ho1—C8—C7	−154.4 (10)	O13—Ho2—N3—C59	144.59 (15)
O4—Ho1—C8—C7	77.9 (10)	O16—Ho2—N3—C59	30.45 (16)
O2—Ho1—C8—C7	−77.3 (9)	O17—Ho2—N3—C59	84.64 (16)
O7—Ho1—C8—C7	9.6 (10)	O10—Ho2—N3—C59	−114.39 (16)
O1—Ho1—C8—C7	112.0 (10)	O11—Ho2—N3—C59	−120.04 (17)
N1—Ho1—C8—C7	3.0 (9)	C40—Ho2—N3—C59	155.22 (16)
O8—Ho1—C8—C7	−149.4 (9)	C48—Ho2—N3—C59	57.36 (16)
C16—Ho1—C8—C7	105.1 (9)	C32—Ho2—N3—C59	−114.62 (16)
C14—C9—C10—C11	1.5 (4)	C67—C68—N4—C64	−2.0 (4)
C15—C9—C10—C11	−178.9 (2)	C65—C64—N4—C68	0.5 (4)
C9—C10—C11—C12	−0.6 (4)	O2—C8—O1—Ho1	8.99 (19)
C10—C11—C12—O6	179.3 (2)	C7—C8—O1—Ho1	−168.92 (17)
C10—C11—C12—C13	−0.4 (4)	O5—Ho1—O1—C8	−159.26 (13)
O6—C12—C13—C14	−179.1 (2)	O1W—Ho1—O1—C8	−8.65 (15)
C11—C12—C13—C14	0.5 (4)	O10—Ho1—O1—C8	−86.61 (12)
C12—C13—C14—C9	0.4 (4)	O4—Ho1—O1—C8	143.95 (13)
C10—C9—C14—C13	−1.4 (4)	O2—Ho1—O1—C8	−5.18 (11)
C15—C9—C14—C13	179.1 (2)	O7—Ho1—O1—C8	123.59 (13)
C10—C9—C15—C16	−97.3 (3)	N1—Ho1—O1—C8	67.42 (12)
C14—C9—C15—C16	82.2 (3)	O8—Ho1—O1—C8	−129.63 (12)
C9—C15—C16—O4	172.8 (2)	C16—Ho1—O1—C8	172.64 (14)
C9—C15—C16—O5	−5.7 (4)	C24—Ho1—O1—C8	175.26 (15)
O5—Ho1—C16—O4	170.4 (2)	O1—C8—O2—Ho1	−9.5 (2)
O1W—Ho1—C16—O4	92.6 (2)	C7—C8—O2—Ho1	168.44 (16)
O10—Ho1—C16—O4	−178.27 (13)	O5—Ho1—O2—C8	35.29 (13)
O2—Ho1—C16—O4	−89.07 (14)	O1W—Ho1—O2—C8	−177.66 (13)
O7—Ho1—C16—O4	64.41 (14)	O10—Ho1—O2—C8	101.33 (12)
O1—Ho1—C16—O4	−91.10 (14)	O4—Ho1—O2—C8	−28.12 (13)
N1—Ho1—C16—O4	−9.71 (15)	O7—Ho1—O2—C8	−131.51 (12)
O8—Ho1—C16—O4	116.29 (14)	O1—Ho1—O2—C8	5.21 (11)
C8—Ho1—C16—O4	−87.82 (14)	N1—Ho1—O2—C8	−92.99 (12)
C24—Ho1—C16—O4	89.77 (14)	O8—Ho1—O2—C8	142.06 (11)
O1W—Ho1—C16—O5	−77.8 (2)	C16—Ho1—O2—C8	2.82 (14)
O10—Ho1—C16—O5	11.32 (13)	C24—Ho1—O2—C8	−175.05 (11)
O4—Ho1—C16—O5	−170.4 (2)	O5—C16—O4—Ho1	9.6 (2)
O2—Ho1—C16—O5	100.52 (13)	C15—C16—O4—Ho1	−168.9 (2)
O7—Ho1—C16—O5	−106.00 (13)	O5—Ho1—O4—C16	−5.42 (12)
O1—Ho1—C16—O5	98.49 (13)	O1W—Ho1—O4—C16	−146.73 (13)
N1—Ho1—C16—O5	179.88 (12)	O10—Ho1—O4—C16	2.14 (16)
O8—Ho1—C16—O5	−54.12 (13)	O2—Ho1—O4—C16	106.44 (14)
C8—Ho1—C16—O5	101.78 (13)	O7—Ho1—O4—C16	−112.14 (14)
C24—Ho1—C16—O5	−80.64 (13)	O1—Ho1—O4—C16	79.16 (14)
C22—C17—C18—C19	0.9 (3)	N1—Ho1—O4—C16	170.26 (15)
C23—C17—C18—C19	179.5 (2)	O8—Ho1—O4—C16	−66.96 (14)
C17—C18—C19—C20	−0.3 (3)	C8—Ho1—O4—C16	94.39 (14)
C18—C19—C20—O9	−179.8 (2)	C24—Ho1—O4—C16	−90.93 (14)
C18—C19—C20—C21	−0.5 (3)	O4—C16—O5—Ho1	−9.7 (2)
O9—C20—C21—C22	−180.0 (2)	C15—C16—O5—Ho1	168.8 (2)
C19—C20—C21—C22	0.7 (3)	O1W—Ho1—O5—C16	151.00 (13)
C20—C21—C22—C17	0.0 (4)	O10—Ho1—O5—C16	−168.39 (14)
C18—C17—C22—C21	−0.7 (3)	O4—Ho1—O5—C16	5.37 (12)
C23—C17—C22—C21	−179.3 (2)	O2—Ho1—O5—C16	−97.02 (13)
C18—C17—C23—C24	−79.4 (3)	O7—Ho1—O5—C16	74.75 (13)
C22—C17—C23—C24	99.1 (3)	O1—Ho1—O5—C16	−72.76 (13)
C17—C23—C24—O7	−46.0 (3)	N1—Ho1—O5—C16	−0.15 (16)
C17—C23—C24—O8	132.9 (2)	O8—Ho1—O5—C16	123.33 (13)
O5—Ho1—C24—O7	−108.77 (14)	C8—Ho1—O5—C16	−81.99 (13)
O1W—Ho1—C24—O7	99.14 (14)	C24—Ho1—O5—C16	99.04 (13)
O10—Ho1—C24—O7	177.81 (14)	O8—C24—O7—Ho1	−8.2 (2)
O4—Ho1—C24—O7	−54.51 (14)	C23—C24—O7—Ho1	170.7 (2)
O2—Ho1—C24—O7	96.49 (16)	O5—Ho1—O7—C24	71.76 (14)
O1—Ho1—C24—O7	−84.1 (2)	O1W—Ho1—O7—C24	−74.04 (14)
N1—Ho1—C24—O7	21.72 (15)	O10—Ho1—O7—C24	−2.46 (16)
O8—Ho1—C24—O7	−172.1 (2)	O4—Ho1—O7—C24	123.22 (15)
C16—Ho1—C24—O7	−81.69 (14)	O2—Ho1—O7—C24	−119.41 (14)
O5—Ho1—C24—O8	63.34 (12)	O1—Ho1—O7—C24	143.10 (13)
O1W—Ho1—C24—O8	−88.75 (12)	N1—Ho1—O7—C24	−157.77 (15)
O10—Ho1—C24—O8	−10.07 (12)	O8—Ho1—O7—C24	4.45 (12)
O4—Ho1—C24—O8	117.61 (12)	C16—Ho1—O7—C24	98.18 (14)
O2—Ho1—C24—O8	−91.39 (14)	C8—Ho1—O7—C24	−164.43 (14)
O7—Ho1—C24—O8	172.1 (2)	O7—C24—O8—Ho2	165.0 (2)
O1—Ho1—C24—O8	88.0 (2)	C23—C24—O8—Ho2	−13.9 (4)
N1—Ho1—C24—O8	−166.16 (11)	Ho1—C24—O8—Ho2	157.3 (3)
C16—Ho1—C24—O8	90.43 (12)	O7—C24—O8—Ho1	7.7 (2)
C30—C25—C26—C27	−0.6 (3)	C23—C24—O8—Ho1	−171.2 (2)
C31—C25—C26—C27	178.0 (2)	O14—Ho2—O8—C24	−82.9 (3)
C25—C26—C27—C28	−0.5 (3)	O2W—Ho2—O8—C24	124.5 (3)
C26—C27—C28—O12	−179.2 (2)	O13—Ho2—O8—C24	−73.9 (3)
C26—C27—C28—C29	1.1 (3)	O16—Ho2—O8—C24	47.6 (3)
O12—C28—C29—C30	179.6 (2)	O17—Ho2—O8—C24	−1.9 (3)
C27—C28—C29—C30	−0.7 (3)	O10—Ho2—O8—C24	−154.8 (3)
C28—C29—C30—C25	−0.4 (4)	O11—Ho2—O8—C24	−171.0 (3)
C26—C25—C30—C29	1.1 (3)	N3—Ho2—O8—C24	77.6 (3)
C31—C25—C30—C29	−177.6 (2)	C40—Ho2—O8—C24	−77.6 (3)
C30—C25—C31—C32	75.6 (3)	C48—Ho2—O8—C24	23.7 (3)
C26—C25—C31—C32	−103.0 (3)	C32—Ho2—O8—C24	−165.6 (3)
C25—C31—C32—O11	11.8 (3)	O14—Ho2—O8—Ho1	72.06 (8)
C25—C31—C32—O10	−167.7 (2)	O2W—Ho2—O8—Ho1	−80.49 (7)
C25—C31—C32—Ho2	−93.5 (7)	O13—Ho2—O8—Ho1	81.09 (6)
O8—Ho2—C32—O11	−168.58 (13)	O16—Ho2—O8—Ho1	−157.47 (7)
O14—Ho2—C32—O11	62.27 (12)	O17—Ho2—O8—Ho1	153.08 (6)
O2W—Ho2—C32—O11	−90.84 (13)	O10—Ho2—O8—Ho1	0.22 (5)
O13—Ho2—C32—O11	116.17 (13)	O11—Ho2—O8—Ho1	−16.04 (8)
O16—Ho2—C32—O11	−95.02 (14)	N3—Ho2—O8—Ho1	−127.40 (10)
O17—Ho2—C32—O11	70.0 (2)	C40—Ho2—O8—Ho1	77.41 (7)
O10—Ho2—C32—O11	168.1 (2)	C48—Ho2—O8—Ho1	178.69 (7)
N3—Ho2—C32—O11	−12.30 (13)	C32—Ho2—O8—Ho1	−10.62 (6)
C40—Ho2—C32—O11	89.18 (13)	O5—Ho1—O8—C24	−112.97 (13)
O8—Ho2—C32—O10	23.30 (12)	O1W—Ho1—O8—C24	82.92 (13)
O14—Ho2—C32—O10	−105.86 (12)	O10—Ho1—O8—C24	168.94 (13)
O2W—Ho2—C32—O10	101.03 (12)	O4—Ho1—O8—C24	−65.46 (13)
O13—Ho2—C32—O10	−51.95 (12)	O2—Ho1—O8—C24	123.49 (13)
O16—Ho2—C32—O10	96.86 (14)	O7—Ho1—O8—C24	−4.32 (12)
O17—Ho2—C32—O10	−98.09 (19)	O1—Ho1—O8—C24	−142.53 (13)
O11—Ho2—C32—O10	−168.1 (2)	N1—Ho1—O8—C24	16.66 (14)
N3—Ho2—C32—O10	179.58 (11)	C16—Ho1—O8—C24	−90.32 (13)
C40—Ho2—C32—O10	−78.94 (12)	C8—Ho1—O8—C24	163.39 (15)
O8—Ho2—C32—C31	−56.9 (7)	O5—Ho1—O8—Ho2	77.86 (6)
O14—Ho2—C32—C31	173.9 (7)	O1W—Ho1—O8—Ho2	−86.25 (7)
O2W—Ho2—C32—C31	20.8 (7)	O10—Ho1—O8—Ho2	−0.23 (5)
O13—Ho2—C32—C31	−132.2 (7)	O4—Ho1—O8—Ho2	125.37 (6)
O16—Ho2—C32—C31	16.6 (8)	O2—Ho1—O8—Ho2	−45.68 (10)
O17—Ho2—C32—C31	−178.3 (6)	O7—Ho1—O8—Ho2	−173.49 (9)
O10—Ho2—C32—C31	−80.2 (7)	O1—Ho1—O8—Ho2	48.30 (12)
O11—Ho2—C32—C31	111.7 (7)	N1—Ho1—O8—Ho2	−152.51 (6)
N3—Ho2—C32—C31	99.4 (7)	C16—Ho1—O8—Ho2	100.51 (7)
C40—Ho2—C32—C31	−159.2 (7)	C8—Ho1—O8—Ho2	−5.78 (16)
C38—C33—C34—C35	−1.0 (3)	C24—Ho1—O8—Ho2	−169.17 (15)
C39—C33—C34—C35	178.8 (2)	O11—C32—O10—Ho1	−151.11 (16)
C33—C34—C35—C36	1.0 (4)	C31—C32—O10—Ho1	28.4 (3)
C34—C35—C36—O15	179.6 (2)	Ho2—C32—O10—Ho1	−139.2 (2)
C34—C35—C36—C37	−0.5 (4)	O11—C32—O10—Ho2	−11.9 (2)
O15—C36—C37—C38	−180.0 (2)	C31—C32—O10—Ho2	167.60 (18)
C35—C36—C37—C38	0.2 (4)	O5—Ho1—O10—C32	53.4 (2)
C36—C37—C38—C33	−0.2 (4)	O1W—Ho1—O10—C32	−148.6 (2)
C34—C33—C38—C37	0.6 (3)	O4—Ho1—O10—C32	47.0 (2)
C39—C33—C38—C37	−179.1 (2)	O2—Ho1—O10—C32	−73.0 (2)
C38—C33—C39—C40	−114.5 (2)	O7—Ho1—O10—C32	141.0 (2)
C34—C33—C39—C40	65.8 (3)	O1—Ho1—O10—C32	−21.0 (2)
C33—C39—C40—O13	−8.7 (3)	N1—Ho1—O10—C32	−105.9 (2)
C33—C39—C40—O14	172.78 (19)	O8—Ho1—O10—C32	135.0 (2)
O8—Ho2—C40—O13	7.79 (13)	C16—Ho1—O10—C32	48.0 (2)
O14—Ho2—C40—O13	178.6 (2)	C8—Ho1—O10—C32	−47.4 (2)
O2W—Ho2—C40—O13	97.85 (18)	C24—Ho1—O10—C32	139.9 (2)
O16—Ho2—C40—O13	−79.15 (13)	O5—Ho1—O10—Ho2	−81.36 (6)
O17—Ho2—C40—O13	−88.42 (12)	O1W—Ho1—O10—Ho2	76.67 (6)
O10—Ho2—C40—O13	72.32 (12)	O4—Ho1—O10—Ho2	−87.76 (8)
O11—Ho2—C40—O13	123.44 (12)	O2—Ho1—O10—Ho2	152.19 (6)
N3—Ho2—C40—O13	−161.29 (12)	O7—Ho1—O10—Ho2	6.19 (8)
C48—Ho2—C40—O13	−86.11 (12)	O1—Ho1—O10—Ho2	−155.73 (6)
C32—Ho2—C40—O13	97.93 (12)	N1—Ho1—O10—Ho2	119.34 (11)
O8—Ho2—C40—O14	−170.86 (12)	O8—Ho1—O10—Ho2	0.22 (4)
O2W—Ho2—C40—O14	−80.79 (19)	C16—Ho1—O10—Ho2	−86.77 (7)
O13—Ho2—C40—O14	−178.6 (2)	C8—Ho1—O10—Ho2	177.87 (7)
O16—Ho2—C40—O14	102.21 (13)	C24—Ho1—O10—Ho2	5.13 (7)
O17—Ho2—C40—O14	92.93 (13)	O8—Ho2—O10—C32	−154.53 (13)
O10—Ho2—C40—O14	−106.32 (13)	O14—Ho2—O10—C32	73.11 (12)
O11—Ho2—C40—O14	−55.20 (13)	O2W—Ho2—O10—C32	−71.53 (12)
N3—Ho2—C40—O14	20.06 (13)	O13—Ho2—O10—C32	125.80 (12)
C48—Ho2—C40—O14	95.25 (13)	O16—Ho2—O10—C32	−118.37 (12)
C32—Ho2—C40—O14	−80.72 (13)	O17—Ho2—O10—C32	139.24 (13)
C46—C41—C42—C43	−0.5 (3)	O11—Ho2—O10—C32	6.53 (11)
C47—C41—C42—C43	177.6 (2)	N3—Ho2—O10—C32	−0.52 (14)
C41—C42—C43—C44	−1.0 (4)	C40—Ho2—O10—C32	99.08 (12)
C42—C43—C44—O18	−178.6 (2)	C48—Ho2—O10—C32	−158.66 (16)
C42—C43—C44—C45	1.6 (4)	O8—Ho2—O10—Ho1	−0.23 (5)
O18—C44—C45—C46	179.5 (2)	O14—Ho2—O10—Ho1	−132.59 (6)
C43—C44—C45—C46	−0.7 (4)	O2W—Ho2—O10—Ho1	82.77 (6)
C44—C45—C46—C41	−0.8 (4)	O13—Ho2—O10—Ho1	−79.90 (6)
C42—C41—C46—C45	1.4 (3)	O16—Ho2—O10—Ho1	35.93 (10)
C47—C41—C46—C45	−176.7 (2)	O17—Ho2—O10—Ho1	−66.46 (12)
C42—C41—C47—C48	−70.4 (3)	O11—Ho2—O10—Ho1	160.83 (9)
C46—C41—C47—C48	107.7 (3)	N3—Ho2—O10—Ho1	153.78 (6)
C41—C47—C48—O16	58.5 (3)	C40—Ho2—O10—Ho1	−106.62 (6)
C41—C47—C48—O17	−120.1 (2)	C48—Ho2—O10—Ho1	−4.37 (18)
O8—Ho2—C48—O16	64.45 (12)	C32—Ho2—O10—Ho1	154.30 (15)
O14—Ho2—C48—O16	−165.14 (11)	O10—C32—O11—Ho2	11.7 (2)
O2W—Ho2—C48—O16	−14.28 (13)	C31—C32—O11—Ho2	−167.7 (2)
O13—Ho2—C48—O16	139.75 (12)	O8—Ho2—O11—C32	12.68 (14)
O17—Ho2—C48—O16	172.2 (2)	O14—Ho2—O11—C32	−112.93 (13)
O10—Ho2—C48—O16	68.2 (2)	O2W—Ho2—O11—C32	80.54 (13)
O11—Ho2—C48—O16	−88.70 (16)	O13—Ho2—O11—C32	−66.80 (13)
N3—Ho2—C48—O16	−93.67 (12)	O16—Ho2—O11—C32	119.09 (13)
C40—Ho2—C48—O16	167.21 (12)	O17—Ho2—O11—C32	−148.98 (12)
O8—Ho2—C48—O17	−107.74 (13)	O10—Ho2—O11—C32	−6.64 (11)
O14—Ho2—C48—O17	22.68 (13)	N3—Ho2—O11—C32	167.56 (13)
O2W—Ho2—C48—O17	173.54 (12)	C40—Ho2—O11—C32	−89.85 (13)
O13—Ho2—C48—O17	−32.44 (13)	C48—Ho2—O11—C32	162.65 (14)
O16—Ho2—C48—O17	−172.2 (2)	O14—C40—O13—Ho2	1.3 (2)
O10—Ho2—C48—O17	−103.94 (18)	C39—C40—O13—Ho2	−177.14 (18)
O11—Ho2—C48—O17	99.12 (16)	O8—Ho2—O13—C40	−172.13 (13)
N3—Ho2—C48—O17	94.14 (13)	O14—Ho2—O13—C40	−0.76 (11)
C40—Ho2—C48—O17	−4.98 (13)	O2W—Ho2—O13—C40	−136.18 (13)
N1—C49—C50—C51	0.3 (3)	O16—Ho2—O13—C40	117.07 (12)
C49—C50—C51—C52	−2.1 (3)	O17—Ho2—O13—C40	83.99 (12)
C49—C50—C51—C56	175.9 (2)	O10—Ho2—O13—C40	−102.80 (12)
C50—C51—C52—C53	1.8 (3)	O11—Ho2—O13—C40	−58.69 (12)
C56—C51—C52—C53	−176.2 (2)	N3—Ho2—O13—C40	23.22 (15)
C51—C52—C53—N1	0.4 (4)	C48—Ho2—O13—C40	98.38 (13)
N2—C54—C55—C56	−0.9 (5)	C32—Ho2—O13—C40	−82.01 (12)
C54—C55—C56—C57	−1.3 (4)	O13—C40—O14—Ho2	−1.3 (2)
C54—C55—C56—C51	175.9 (3)	C39—C40—O14—Ho2	177.18 (16)
C50—C51—C56—C57	27.4 (4)	O8—Ho2—O14—C40	11.52 (15)
C52—C51—C56—C57	−154.7 (3)	O2W—Ho2—O14—C40	142.61 (12)
C50—C51—C56—C55	−149.7 (3)	O13—Ho2—O14—C40	0.76 (12)
C52—C51—C56—C55	28.2 (4)	O16—Ho2—O14—C40	−98.44 (13)
C55—C56—C57—C58	2.2 (4)	O17—Ho2—O14—C40	−80.08 (13)
C51—C56—C57—C58	−175.1 (3)	O10—Ho2—O14—C40	72.46 (13)
C56—C57—C58—N2	−1.0 (5)	O11—Ho2—O14—C40	120.84 (13)
N3—C59—C60—C61	−1.4 (4)	N3—Ho2—O14—C40	−159.75 (14)
C59—C60—C61—C62	3.9 (3)	C48—Ho2—O14—C40	−90.28 (13)
C59—C60—C61—C66	−173.4 (2)	C32—Ho2—O14—C40	97.35 (13)
C60—C61—C62—C63	−2.8 (3)	O17—C48—O16—Ho2	7.9 (2)
C66—C61—C62—C63	174.4 (2)	C47—C48—O16—Ho2	−170.79 (17)
C61—C62—C63—N3	−0.8 (4)	O8—Ho2—O16—C48	−113.83 (12)
N4—C64—C65—C66	1.4 (5)	O14—Ho2—O16—C48	18.33 (14)
C64—C65—C66—C67	−1.9 (4)	O2W—Ho2—O16—C48	165.51 (13)
C64—C65—C66—C61	174.4 (2)	O13—Ho2—O16—C48	−45.96 (13)
C60—C61—C66—C67	159.2 (2)	O17—Ho2—O16—C48	−4.39 (11)
C62—C61—C66—C67	−17.9 (3)	O10—Ho2—O16—C48	−147.22 (11)
C60—C61—C66—C65	−16.9 (3)	O11—Ho2—O16—C48	127.57 (12)
C62—C61—C66—C65	166.0 (2)	N3—Ho2—O16—C48	79.33 (12)
C65—C66—C67—C68	0.6 (4)	C40—Ho2—O16—C48	−15.63 (14)
C61—C66—C67—C68	−175.8 (2)	C32—Ho2—O16—C48	169.57 (11)
C66—C67—C68—N4	1.4 (5)	O16—C48—O17—Ho2	−7.6 (2)
C52—C53—N1—C49	−2.2 (3)	C47—C48—O17—Ho2	171.07 (17)
C52—C53—N1—Ho1	172.73 (19)	O8—Ho2—O17—C48	74.69 (13)
C50—C49—N1—C53	1.9 (3)	O14—Ho2—O17—C48	−157.25 (13)
C50—C49—N1—Ho1	−172.80 (17)	O2W—Ho2—O17—C48	−7.99 (15)
O5—Ho1—N1—C53	41.12 (19)	O13—Ho2—O17—C48	146.66 (13)
O1W—Ho1—N1—C53	−122.84 (17)	O16—Ho2—O17—C48	4.33 (11)
O10—Ho1—N1—C53	−165.32 (14)	O10—Ho2—O17—C48	133.17 (12)
O4—Ho1—N1—C53	36.50 (16)	O11—Ho2—O17—C48	−122.03 (13)
O2—Ho1—N1—C53	160.60 (17)	N3—Ho2—O17—C48	−78.82 (13)
O7—Ho1—N1—C53	−43.12 (16)	C40—Ho2—O17—C48	174.91 (14)
O1—Ho1—N1—C53	108.75 (17)	C32—Ho2—O17—C48	−165.17 (15)

### Hydrogen-bond geometry (Å, °)

**Table:**

*D*—H···*A*	*D*—H	H···*A*	*D*···*A*	*D*—H···*A*
O6—H6B···O3W^i^	0.82	1.86	2.646 (3)	160
O9—H9A···O17^ii^	0.82	1.86	2.676 (2)	173
O12—H12A···O11^iii^	0.82	1.94	2.748 (2)	168
O15—H15C···O6^iv^	0.82	1.90	2.714 (2)	174
O18—H18B···O9^i^	0.82	1.95	2.769 (3)	174
O1W—H1WA···O13	0.84 (4)	1.95 (2)	2.736 (2)	156 (3)
O1W—H1WB···N4^v^	0.83 (2)	1.97 (2)	2.781 (3)	167 (4)
O2W—H2WA···O5	0.84 (4)	1.97 (2)	2.735 (2)	150 (3)
O3W—H3WA···O1^vi^	0.84 (2)	1.95 (2)	2.780 (2)	176 (4)
O2W—H2WB···N2^i^	0.83 (2)	2.02 (2)	2.835 (3)	166 (4)
O3—H3B···O12^v^	0.82	2.05	2.735 (3)	141
O3W—H3WB···O3	0.83 (4)	2.00 (4)	2.803 (3)	164 (4)

Symmetry codes: (i) *x*, *y*−1, *z*; (ii) −*x*, −*y*+1, −*z*; (iii) −*x*, −*y*, −*z*+1; (iv) *x*−1, *y*+1, *z*; (v) *x*, *y*+1, *z*; (vi) −*x*+1, −*y*+1, −*z*+1.

## References

[bb1] Bruker (2006). *APEX2* and *SAINT* Bruker AXS Inc., Madison, Wisconsin, USA.

[bb2] Fang, R.-Q. & Zhang, X.-M. (2006). *Inorg. Chem.* **45**, 4801–4810. 10.1021/ic052099m 16749845

[bb3] Liu, J.-L., Li, H.-Q. & Zhao, G.-L. (2010). *Acta Cryst.* E**66**, m9.

[bb4] Munoz, J. C., Atria, A. M., Baggio, R., Garland, M. T. & Orrego, C. (2005). *Inorg. Chim. Acta*, **358**, 4027–4033.

[bb5] Sheldrick, G. M. (1996). *SADABS* University of Göttingen, Germany.

[bb6] Sheldrick, G. M. (2008). *Acta Cryst.* A**64**, 112–122. 10.1107/S0108767307043930 18156677

[bb7] Wang, G.-H., Lei, Y.-Q. & Wang, N. (2010). *Cryst. Growth Des.* **10**, 4060-4067.

[bb8] Wang, X.-X. & Sevov, S. (2008). *Inorg. Chem.* **47**, 1037–1043. 10.1021/ic701893z 18095675

